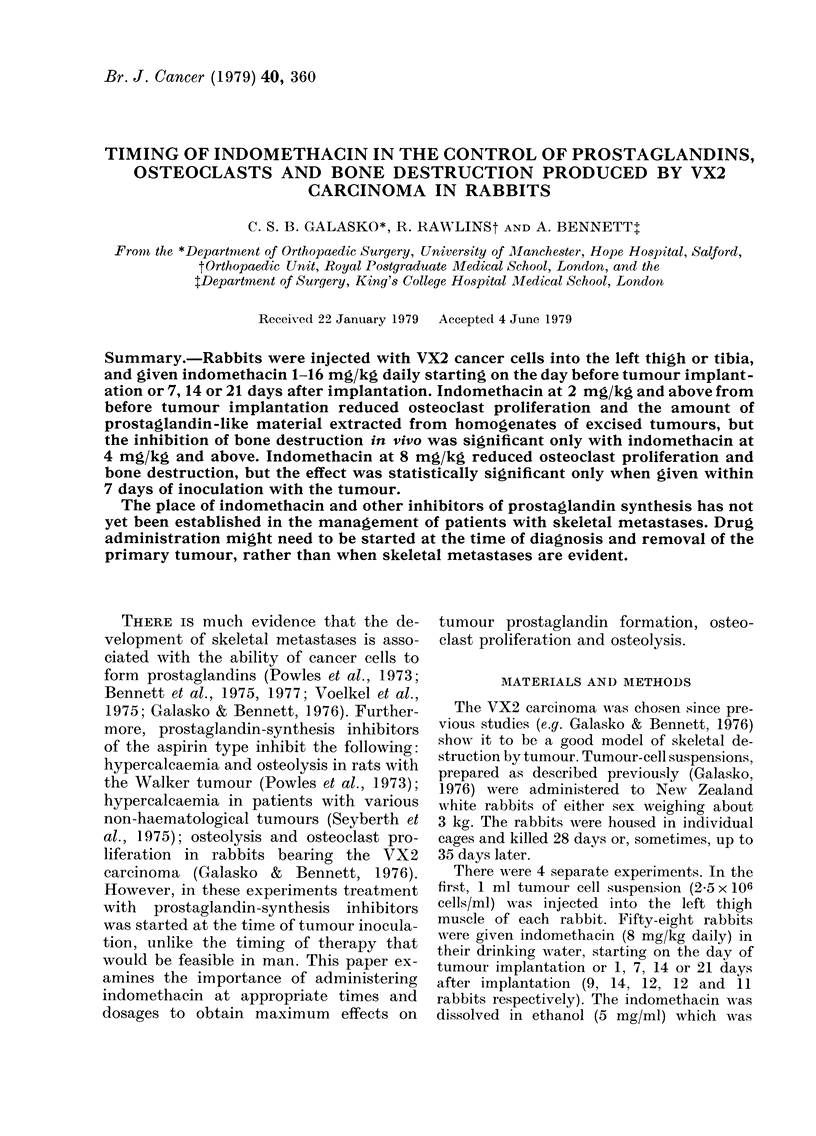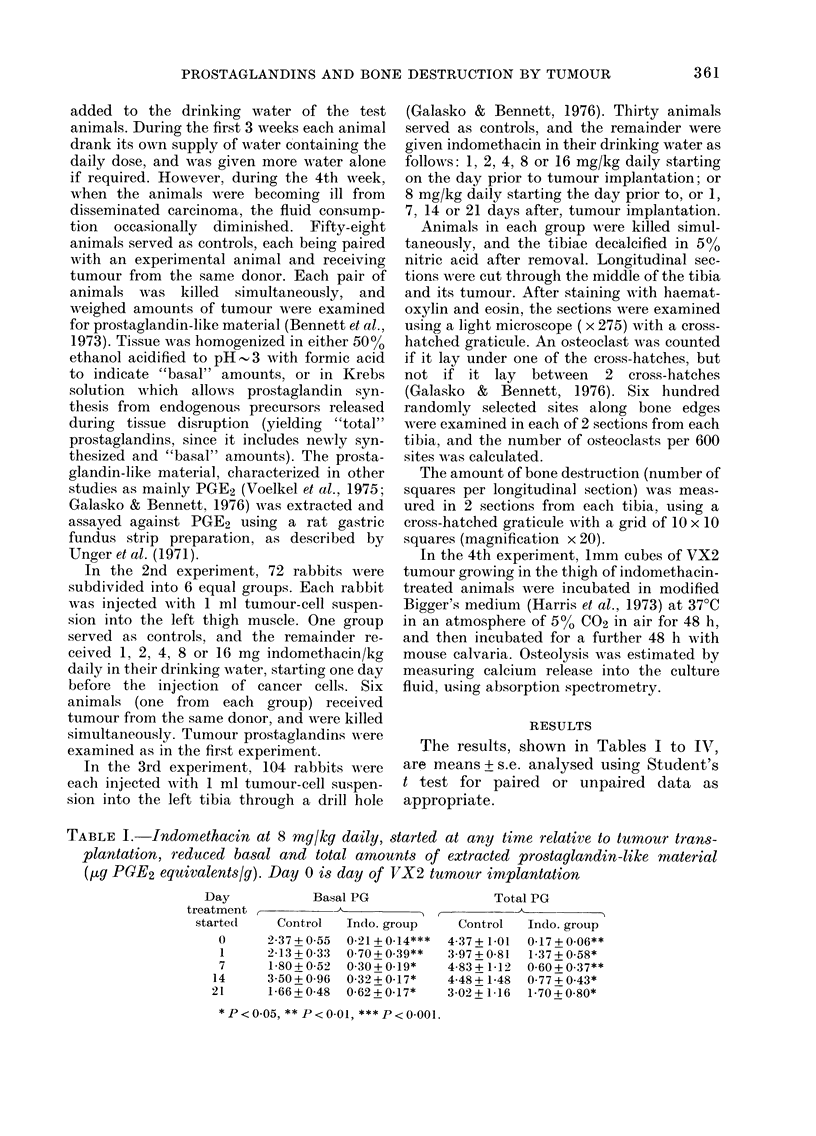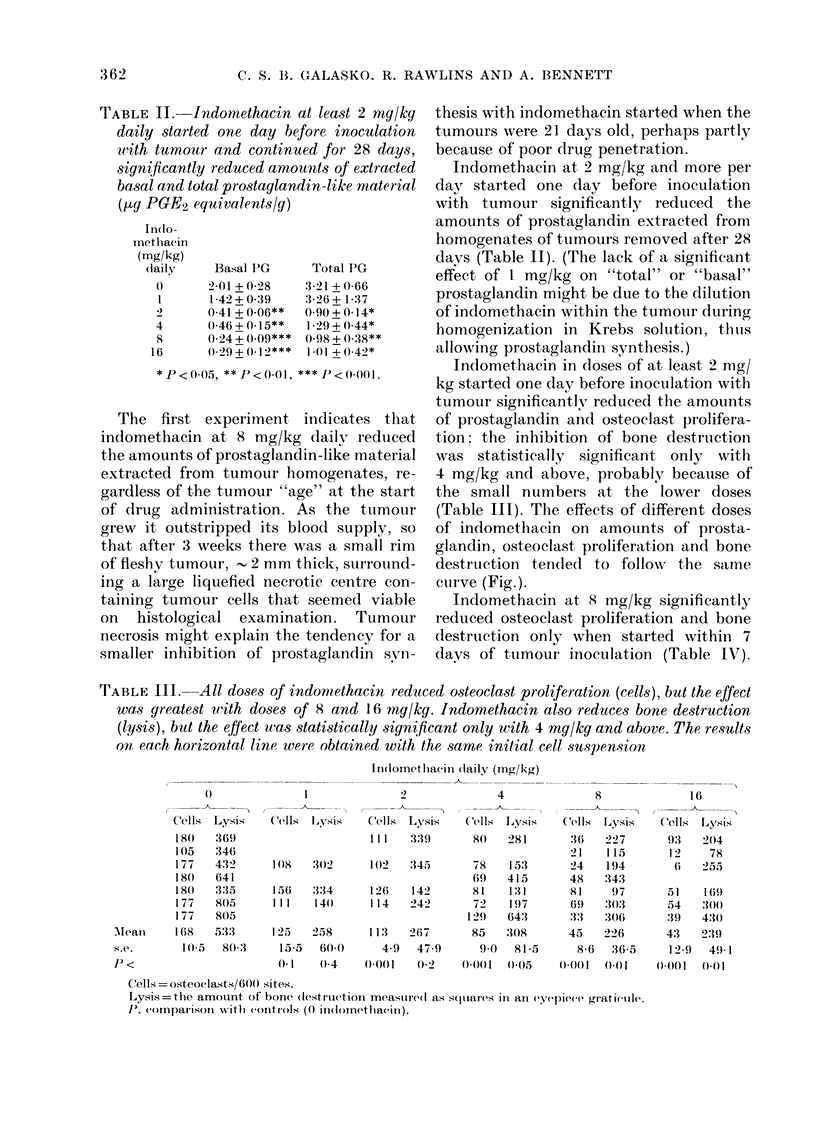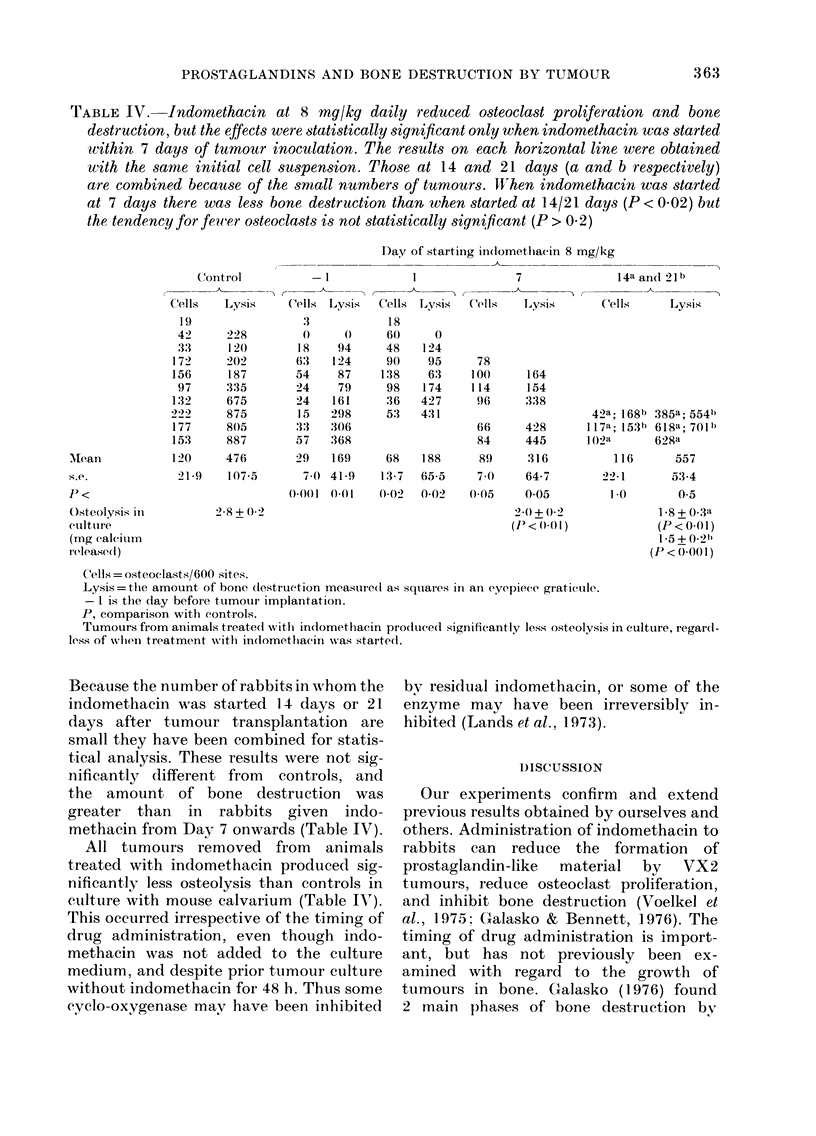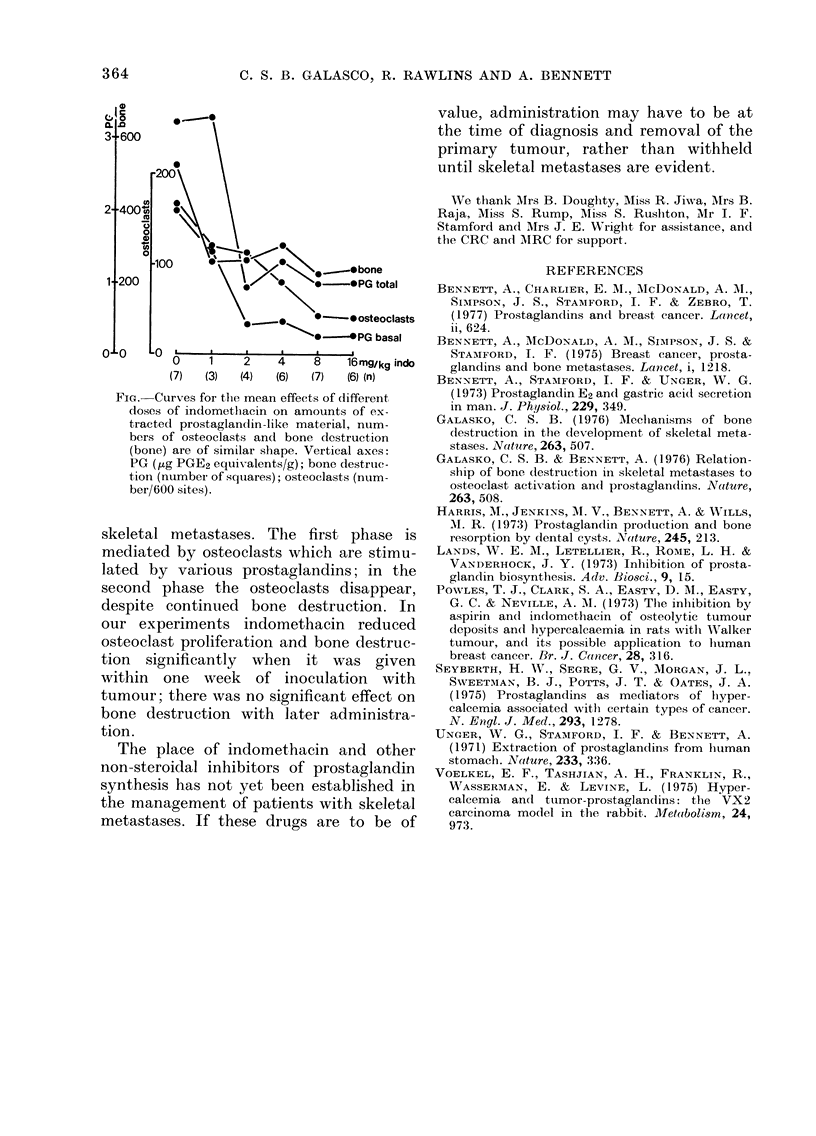# Timing of indomethacin in the control of prostaglandins, osteoclasts and bone destruction produced by VX2 carcinoma in rabbits.

**DOI:** 10.1038/bjc.1979.189

**Published:** 1979-09

**Authors:** C. S. Galasko, R. Rawlins, A. Bennett

## Abstract

Rabbits were injected with VX2 cancer cells into the left thigh or tibia, and given indomethacin 1-16 mg/kg daily starting on the day before tumour implantation or 7, 14 or 21 days after implantation. Indomethacin at 2 mg/kg and above from before tumour implantation reduced osteoclast proliferation and the amount of prostaglandin-like material extracted from homogenates of excised tumours, but the inhibition of bone destruction in vivo was significant only with indomethacin at 4 mg/kg and above. Indomethacin at 8 mg/kg reduced osteoclast proliferation and bone destruction, but the effect was statistically significant only when given within 7 days of inoculation with the tumour. The place of indomethacin and other inhibitors of prostaglandin synthesis has not yet been established in the management of patients with skeletal metastases. Drug administration might need to be started at the time of diagnosis and removal of the primary tumour, rather than when skeletal metastases are evident.


*Br. J. Cancer* (1979) **40**, 360

### TIMING OF INDOMETHACIN IN THE CONTROL OF PROSTAGLANDINS, OSTEOCLASTS AND BONE DESTRUCTION PRODUCED BY VX2 CARCINOMA IN RABBITS

C. S. B. GALASKO*, R. RAWLINS† AND A. BENNETT‡

*From the *Department of Orthopaedic Surgery, University of Manchester, Hope Hospital, Salford, †Orthopaedic Unit, Royal Postgraduate Medical School, London, and the ‡Department of Surgery, King's College Hospital Medical School, London*

Received 22 January 1979 Accepted 4 June 1979

**Summary.—Rabbits were injected with VX2 cancer cells into the left thigh or tibia, and given indomethacin 1–16 mg/kg daily starting on the day before tumour implantation or 7, 14 or 21 days after implantation. Indomethacin at 2 mg/kg and above from before tumour implantation reduced osteoclast proliferation and the amount of prostaglandin-like material extracted from homogenates of excised tumours, but the inhibition of bone destruction *in vivo* was significant only with indomethacin at 4 mg/kg and above. Indomethacin at 8 mg/kg reduced osteoclast proliferation and bone destruction, but the effect was statistically significant only when given within 7 days of inoculation with the tumour.**

**The place of indomethacin and other inhibitors of prostaglandin synthesis has not yet been established in the management of patients with skeletal metastases. Drug administration might need to be started at the time of diagnosis and removal of the primary tumour, rather than when skeletal metastases are evident.**

THERE IS much evidence that the development of skeletal metastases is associated with the ability of cancer cells to form prostaglandins (Powles *et al.*, 1973; Bennett *et al.*, 1975, 1977; Voelkel *et al.*, 1975; Galasko & Bennett, 1976). Furthermore, prostaglandin-synthesis inhibitors of the aspirin type inhibit the following: hypercalcaemia and osteolysis in rats with the Walker tumour (Powles *et al.*, 1973); hypercalcaemia in patients with various non-haematological tumours (Seyberth *et al.*, 1975); osteolysis and osteoclast proliferation in rabbits bearing the VX2 carcinoma (Galasko & Bennett, 1976). However, in these experiments treatment with prostaglandin-synthesis inhibitors was started at the time of tumour inoculation, unlike the timing of therapy that would be feasible in man. This paper examines the importance of administering indomethacin at appropriate times and dosages to obtain maximum effects on tumour prostaglandin formation, osteoclast proliferation and osteolysis.

### MATERIALS AND METHODS

The VX2 carcinoma was chosen since previous studies (*e.g.* Galasko & Bennett, 1976) show it to be a good model of skeletal destruction by tumour. Tumour-cell suspensions, prepared as described previously (Galasko, 1976) were administered to New Zealand white rabbits of either sex weighing about 3 kg. The rabbits were housed in individual cages and killed 28 days or, sometimes, up to 35 days later.

There were 4 separate experiments. In the first, 1 ml tumour cell suspension ($2{\cdot}5 \times 10^6$ cells/ml) was injected into the left thigh muscle of each rabbit. Fifty-eight rabbits were given indomethacin (8 mg/kg daily) in their drinking water, starting on the day of tumour implantation or 1, 7, 14 or 21 days after implantation (9, 14, 12, 12 and 11 rabbits respectively). The indomethacin was dissolved in ethanol (5 mg/ml) which was added to the drinking water of the test animals. During the first 3 weeks each animal drank its own supply of water containing the daily dose, and was given more water alone if required. However, during the 4th week, when the animals were becoming ill from disseminated carcinoma, the fluid consumption occasionally diminished. Fifty-eight animals served as controls, each being paired with an experimental animal and receiving tumour from the same donor. Each pair of animals was killed simultaneously, and weighed amounts of tumour were examined for prostaglandin-like material (Bennett *et al.*, 1973). Tissue was homogenized in either 50% ethanol acidified to pH $\sim$3 with formic acid to indicate "basal" amounts, or in Krebs solution which allows prostaglandin synthesis from endogenous precursors released during tissue disruption (yielding "total" prostaglandins, since it includes newly synthesized and "basal" amounts). The prostaglandin-like material, characterized in other studies as mainly $PGE_2$ (Voelkel *et al.*, 1975; Galasko & Bennett, 1976) was extracted and assayed against $PGE_2$ using a rat gastric fundus strip preparation, as described by Unger *et al.* (1971).

In the 2nd experiment, 72 rabbits were subdivided into 6 equal groups. Each rabbit was injected with 1 ml tumour-cell suspension into the left thigh muscle. One group served as controls, and the remainder received 1, 2, 4, 8 or 16 mg indomethacin/kg daily in their drinking water, starting one day before the injection of cancer cells. Six animals (one from each group) received tumour from the same donor, and were killed simultaneously. Tumour prostaglandins were examined as in the first experiment.

In the 3rd experiment, 104 rabbits were each injected with 1 ml tumour-cell suspension into the left tibia through a drill hole (Galasko & Bennett, 1976). Thirty animals served as controls, and the remainder were given indomethacin in their drinking water as follows: 1, 2, 4, 8 or 16 mg/kg daily starting on the day prior to tumour implantation; or 8 mg/kg daily starting the day prior to, or 1, 7, 14 or 21 days after, tumour implantation.

Animals in each group were killed simultaneously, and the tibiae decalcified in 5% nitric acid after removal. Longitudinal sections were cut through the middle of the tibia and its tumour. After staining with haematoxylin and eosin, the sections were examined using a light microscope ($\times$275) with a cross-hatched graticule. An osteoclast was counted if it lay under one of the cross-hatches, but not if it lay between 2 cross-hatches (Galasko & Bennett, 1976). Six hundred randomly selected sites along bone edges were examined in each of 2 sections from each tibia, and the number of osteoclasts per 600 sites was calculated.

The amount of bone destruction (number of squares per longitudinal section) was measured in 2 sections from each tibia, using a cross-hatched graticule with a grid of 10$\times$10 squares (magnification $\times$20).

In the 4th experiment, 1mm cubes of VX2 tumour growing in the thigh of indomethacin-treated animals were incubated in modified Bigger's medium (Harris *et al.*, 1973) at 37°C in an atmosphere of 5% $CO_2$ in air for 48 h, and then incubated for a further 48 h with mouse calvaria. Osteolysis was estimated by measuring calcium release into the culture fluid, using absorption spectrometry.

### RESULTS

The results, shown in Tables I to IV, are means ± s.e. analysed using Student's *t* test for paired or unpaired data as appropriate.

TABLE I.—*Indomethacin at 8 mg/kg daily, started at any time relative to tumour transplantation, reduced basal and total amounts of extracted prostaglandin-like material (μg $PGE_2$ equivalents/g). Day 0 is day of VX2 tumour implantation*

| Day treatment started | Basal PG | | Total PG | |
|---|---|---|---|---|
| | Control | Indo. group | Control | Indo. group |
| 0 | 2·37 ± 0·55 | 0·21 ± 0·14*** | 4·37 ± 1·01 | 0·17 ± 0·06** |
| 1 | 2·13 ± 0·33 | 0·70 ± 0·39** | 3·97 ± 0·81 | 1·37 ± 0·58* |
| 7 | 1·80 ± 0·52 | 0·30 ± 0·19* | 4·83 ± 1·12 | 0·60 ± 0·37** |
| 14 | 3·50 ± 0·96 | 0·32 ± 0·17* | 4·48 ± 1·48 | 0·77 ± 0·43* |
| 21 | 1·66 ± 0·48 | 0·62 ± 0·17* | 3·02 ± 1·16 | 1·70 ± 0·80* |

* $P < 0·05$, ** $P < 0·01$, *** $P < 0·001$.

TABLE II.—*Indomethacin at least 2 mg/kg daily started one day before inoculation with tumour and continued for 28 days, significantly reduced amounts of extracted basal and total prostaglandin-like material ($\mu g$ $PGE_2$ equivalents/g)*

| Indomethacin (mg/kg) daily | Basal PG | Total PG |
|---|---|---|
| 0 | 2·01 ± 0·28 | 3·21 ± 0·66 |
| 1 | 1·42 ± 0·39 | 3·26 ± 1·37 |
| 2 | 0·41 ± 0·06** | 0·90 ± 0·14* |
| 4 | 0·46 ± 0·15** | 1·29 ± 0·44* |
| 8 | 0·24 ± 0·09*** | 0·98 ± 0·38** |
| 16 | 0·29 ± 0·12*** | 1·01 ± 0·42* |

The first experiment indicates that indomethacin at 8 mg/kg daily reduced the amounts of prostaglandin-like material extracted from tumour homogenates, regardless of the tumour "age" at the start of drug administration. As the tumour grew it outstripped its blood supply, so that after 3 weeks there was a small rim of fleshy tumour, ~ 2 mm thick, surrounding a large liquefied necrotic centre containing tumour cells that seemed viable on histological examination. Tumour necrosis might explain the tendency for a smaller inhibition of prostaglandin synthesis with indomethacin started when the tumours were 21 days old, perhaps partly because of poor drug penetration.

Indomethacin at 2 mg/kg and more per day started one day before inoculation with tumour significantly reduced the amounts of prostaglandin extracted from homogenates of tumours removed after 28 days (Table II). (The lack of a significant effect of 1 mg/kg on "total" or "basal" prostaglandin might be due to the dilution of indomethacin within the tumour during homogenization in Krebs solution, thus allowing prostaglandin synthesis.)

Indomethacin in doses of at least 2 mg/kg started one day before inoculation with tumour significantly reduced the amounts of prostaglandin and osteoclast proliferation; the inhibition of bone destruction was statistically significant only with 4 mg/kg and above, probably because of the small numbers at the lower doses (Table III). The effects of different doses of indomethacin on amounts of prostaglandin, osteoclast proliferation and bone destruction tended to follow the same curve (Fig.).

Indomethacin at 8 mg/kg significantly reduced osteoclast proliferation and bone destruction only when started within 7 days of tumour inoculation (Table IV).

TABLE III.—*All doses of indomethacin reduced osteoclast proliferation (cells), but the effect was greatest with doses of 8 and 16 mg/kg. Indomethacin also reduces bone destruction (lysis), but the effect was statistically significant only with 4 mg/kg and above. The results on each horizontal line were obtained with the same initial cell suspension*

| | Indomethacin daily (mg/kg) | | | | | | | | | | | |
|---|---|---|---|---|---|---|---|---|---|---|---|---|
| | 0 | | 1 | | 2 | | 4 | | 8 | | 16 | |
| | Cells | Lysis | Cells | Lysis | Cells | Lysis | Cells | Lysis | Cells | Lysis | Cells | Lysis |
| | 180 | 369 | | | 111 | 339 | 80 | 281 | 36 | 227 | 93 | 204 |
| | 105 | 346 | | | | | | | 21 | 115 | 12 | 78 |
| | 177 | 432 | 108 | 302 | 102 | 345 | 78 | 153 | 24 | 194 | 6 | 255 |
| | 180 | 641 | | | | | 69 | 415 | 48 | 343 | | |
| | 180 | 335 | 156 | 334 | 126 | 142 | 81 | 131 | 81 | 97 | 51 | 169 |
| | 177 | 805 | 111 | 140 | 114 | 242 | 72 | 197 | 69 | 303 | 54 | 300 |
| | 177 | 805 | | | | | 129 | 643 | 33 | 306 | 39 | 430 |
| Mean | 168 | 533 | 125 | 258 | 113 | 267 | 85 | 308 | 45 | 226 | 43 | 239 |
| s.e. | 10·5 | 80·3 | 15·5 | 60·0 | 4·9 | 47·9 | 9·0 | 81·5 | 8·6 | 36·5 | 12·9 | 49·1 |
| $P <$ | | | 0·1 | 0·4 | 0·001 | 0·2 | 0·001 | 0·05 | 0·001 | 0·01 | 0·001 | 0·01 |

Cells = osteoclasts/600 sites.
Lysis = the amount of bone destruction measured as squares in an eyepiece graticule.
$P$, comparison with controls (0 indomethacin).

TABLE IV.—*Indomethacin at 8 mg/kg daily reduced osteoclast proliferation and bone destruction, but the effects were statistically significant only when indomethacin was started within 7 days of tumour inoculation. The results on each horizontal line were obtained with the same initial cell suspension. Those at 14 and 21 days (a and b respectively) are combined because of the small numbers of tumours. When indomethacin was started at 7 days there was less bone destruction than when started at 14/21 days ($P < 0{\cdot}02$) but the tendency for fewer osteoclasts is not statistically significant ($P > 0{\cdot}2$)*

| | Control | | Day of starting indomethacin 8 mg/kg: −1 | | 1 | | 7 | | 14[a] and 21[b] | |
|---|---|---|---|---|---|---|---|---|---|---|
| | Cells | Lysis | Cells | Lysis | Cells | Lysis | Cells | Lysis | Cells | Lysis |
| | 19 | | 3 | | 18 | | | | | |
| | 42 | 228 | 0 | 0 | 60 | 0 | | | | |
| | 33 | 120 | 18 | 94 | 48 | 124 | | | | |
| | 172 | 202 | 63 | 124 | 90 | 95 | 78 | | | |
| | 156 | 187 | 54 | 87 | 138 | 63 | 100 | 164 | | |
| | 97 | 335 | 24 | 79 | 98 | 174 | 114 | 154 | | |
| | 132 | 675 | 24 | 161 | 36 | 427 | 96 | 338 | | |
| | 222 | 875 | 15 | 298 | 53 | 431 | | | 42[a]; 168[b] | 385[a]; 554[b] |
| | 177 | 805 | 33 | 306 | | | 66 | 428 | 117[a]; 153[b] | 618[a]; 701[b] |
| | 153 | 887 | 57 | 368 | | | 84 | 445 | 102[a] | 628[a] |
| Mean | 120 | 476 | 29 | 169 | 68 | 188 | 89 | 316 | 116 | 557 |
| s.e. | 21·9 | 107·5 | 7·0 | 41·9 | 13·7 | 65·5 | 7·0 | 64·7 | 22·1 | 53·4 |
| $P <$ | | | 0·001 | 0·01 | 0·02 | 0·02 | 0·05 | 0·05 | 1·0 | 0·5 |
| Osteolysis in culture (mg calcium released) | | $2{\cdot}8 \pm 0{\cdot}2$ | | | | | | $2{\cdot}0 \pm 0{\cdot}2$ ($P < 0{\cdot}01$) | | $1{\cdot}8 \pm 0{\cdot}3$[a] ($P < 0{\cdot}01$)<br>$1{\cdot}5 \pm 0{\cdot}2$[b] ($P < 0{\cdot}001$) |

Cells = osteoclasts/600 sites.
Lysis = the amount of bone destruction measured as squares in an eyepiece graticule.
− 1 is the day before tumour implantation.
$P$, comparison with controls.
Tumours from animals treated with indomethacin produced significantly less osteolysis in culture, regardless of when treatment with indomethacin was started.

Because the number of rabbits in whom the indomethacin was started 14 days or 21 days after tumour transplantation are small they have been combined for statistical analysis. These results were not significantly different from controls, and the amount of bone destruction was greater than in rabbits given indomethacin from Day 7 onwards (Table IV).

All tumours removed from animals treated with indomethacin produced significantly less osteolysis than controls in culture with mouse calvarium (Table IV). This occurred irrespective of the timing of drug administration, even though indomethacin was not added to the culture medium, and despite prior tumour culture without indomethacin for 48 h. Thus some cyclo-oxygenase may have been inhibited by residual indomethacin, or some of the enzyme may have been irreversibly inhibited (Lands *et al.*, 1973).

### DISCUSSION

Our experiments confirm and extend previous results obtained by ourselves and others. Administration of indomethacin to rabbits can reduce the formation of prostaglandin-like material by VX2 tumours, reduce osteoclast proliferation, and inhibit bone destruction (Voelkel *et al.*, 1975; Galasko & Bennett, 1976). The timing of drug administration is important, but has not previously been examined with regard to the growth of tumours in bone. Galasko (1976) found 2 main phases of bone destruction by

FIG.—Curves for the mean effects of different doses of indomethacin on amounts of extracted prostaglandin-like material, numbers of osteoclasts and bone destruction (bone) are of similar shape. Vertical axes: PG ($\mu$g $PGE_2$ equivalents/g); bone destruction (number of squares); osteoclasts (number/600 sites).

skeletal metastases. The first phase is mediated by osteoclasts which are stimulated by various prostaglandins; in the second phase the osteoclasts disappear, despite continued bone destruction. In our experiments indomethacin reduced osteoclast proliferation and bone destruction significantly when it was given within one week of inoculation with tumour; there was no significant effect on bone destruction with later administration.

The place of indomethacin and other non-steroidal inhibitors of prostaglandin synthesis has not yet been established in the management of patients with skeletal metastases. If these drugs are to be of value, administration may have to be at the time of diagnosis and removal of the primary tumour, rather than withheld until skeletal metastases are evident.

We thank Mrs B. Doughty, Miss R. Jiwa, Mrs B. Raja, Miss S. Rump, Miss S. Rushton, Mr I. F. Stamford and Mrs J. E. Wright for assistance, and the CRC and MRC for support.

### REFERENCES

BENNETT, A., CHARLIER, E. M., MCDONALD, A. M., SIMPSON, J. S., STAMFORD, I. F. & ZEBRO, T. (1977) Prostaglandins and breast cancer. *Lancet*, ii, 624.

BENNETT, A., MCDONALD, A. M., SIMPSON, J. S. & STAMFORD, I. F. (1975) Breast cancer, prostaglandins and bone metastases. *Lancet*, i, 1218.

BENNETT, A., STAMFORD, I. F. & UNGER, W. G. (1973) Prostaglandin $E_2$ and gastric acid secretion in man. *J. Physiol.*, **229**, 349.

GALASKO, C. S. B. (1976) Mechanisms of bone destruction in the development of skeletal metastases. *Nature*, **263**, 507.

GALASKO, C. S. B. & BENNETT, A. (1976) Relationship of bone destruction in skeletal metastases to osteoclast activation and prostaglandins. *Nature*, **263**, 508.

HARRIS, M., JENKINS, M. V., BENNETT, A. & WILLS, M. R. (1973) Prostaglandin production and bone resorption by dental cysts. *Nature*, **245**, 213.

LANDS, W. E. M., LETELLIER, R., ROME, L. H. & VANDERHOCK, J. Y. (1973) Inhibition of prostaglandin biosynthesis. *Adv. Biosci.*, **9**, 15.

POWLES, T. J., CLARK, S. A., EASTY, D. M., EASTY, G. C. & NEVILLE, A. M. (1973) The inhibition by aspirin and indomethacin of osteolytic tumour deposits and hypercalcaemia in rats with Walker tumour, and its possible application to human breast cancer. *Br. J. Cancer*, **28**, 316.

SEYBERTH, H. W., SEGRE, G. V., MORGAN, J. L., SWEETMAN, B. J., POTTS, J. T. & OATES, J. A. (1975) Prostaglandins as mediators of hypercalcemia associated with certain types of cancer. *N. Engl. J. Med.*, **293**, 1278.

UNGER, W. G., STAMFORD, I. F. & BENNETT, A. (1971) Extraction of prostaglandins from human stomach. *Nature*, **233**, 336.

VOELKEL, E. F., TASHJIAN, A. H., FRANKLIN, R., WASSERMAN, E. & LEVINE, L. (1975) Hypercalcemia and tumor-prostaglandins: the VX2 carcinoma model in the rabbit. *Metabolism*, **24**, 973.